# Is Plant Microbiota a Driver of Resistance to the Vector-Borne Pathogen *Xylella fastidiosa*?

**DOI:** 10.3390/pathogens11121492

**Published:** 2022-12-08

**Authors:** Alejandro Cabezas-Cruz, Apolline Maitre

**Affiliations:** 1 ANSES, INRAE, Ecole Nationale Vétérinaire d’Alfort, UMR BIPAR, Laboratoire de Santé Animale, F-94700 Maisons-Alfort, France; 2 INRAE, UR 0045 Laboratoire de Recherches Sur Le Développement de L’Elevage (SELMET-LRDE), 20250 Corte, France; 3 EA 7310, Laboratoire de Virologie, Université de Corse, 20250 Corte, France

*Xylella fastidiosa* is a vector-borne plant vascular bacterial pathogen that causes several economically important diseases, including Pierce’s disease (PD) in grapevine and olive quick decline syndrome (OQDS) in olive trees, among others [1]. The severity and timing of symptoms produced by *X. fastidiosa* differ among olive cultivars in Italy, with ‘Cellina di Nardò’ being one of the most severely affected cultivars, while other cultivars such as ‘Leccino’ exhibit milder symptoms and have been regarded as resistant [2]. Depending on the host plant, *X. fastidiosa* can establish non-symptomatic associations as a commensal endophyte [1,3]. In fact, in the majority of plant hosts, *X. fastidiosa* does not cause severe disease [4], and recent discoveries indicate that, even in its parasitic form, the bacterium displays the hallmarks of a commensal lifestyle [3]. What drives the transitions of *X. fastidiosa* along the ‘*parasite–mutualist continuum*’ [5]? Resistance in the ‘Leccino’ olive trees has been associated with the amount of lignin [6] or secondary metabolites, such as hydroxytyrosol glucoside [7], produced by this cultivar. In grapevine plants, immunity to the *X. fastidiosa* O-antigen was found to dictate the type of association of the bacteria with the host plant as a commensal or a parasite [3,8]. In addition, genetic factors such as gene gain/loss, recombination, genetic diversity, and linkage disequilibrium could also influence the host specificity and pathogenicity of *X. fastidiosa* [9,10]. However, drivers of *X. fastidiosa* virulence and/or plant-resistant traits are not completely understood. Are balanced plant–pathogen interactions enough to explain the changes in *X. fastidiosa* pathogenicity across plant hosts? Can other factors such as plant microbiota counterbalance plant resistance and/or *X. fastidiosa* commensal lifestyles?

A recent research paper by Vergine et al. [11] attempts to answer these questions. In their study, major differences in the bacterial and fungal microbiota of *X. fastidiosa*-infected and -uninfected olive trees of the ‘Leccino’ and ‘Cellina di Nardò’ cultivars were found [11]. Variations in microbiota composition can drive pathogen colonization resistance in animals [12] and plants [13]. Microbiota evolved complex mechanisms to reduce pathogen growth, including nutrient competition, competitive metabolic interactions, niche exclusion, and the induction of host immune response [11,13]. For example, in the flowering plant *Catharanthus roseus*, the endophyte *Curtobacterium flaccumfaciens* inhibited the growth of *X. fastidiosa* in vitro and reduced the symptoms caused by this bacterium to the plant host [14]. It can be challenging to identify beneficial plant-associated microbes with antagonistic activity against *X. fastidiosa* [15], but it is crucial to develop novel control methods against diseases caused by these bacteria [16]. In their work, Vergine et al. [11] identified some taxa found predominantly in the ‘Leccino’ cultivar which were proposed to be potentially involved in the resistance of cultivar to *X. fastidiosa*. Among them was an unidentified member of the Burkholderiaceae family. Within this bacterial family, there are some other species (e.g., *Paraburkholderia phytofirmans* strain PsJN) with strong activity against *X. fastidiosa* [17]. Furthermore, network analysis, a powerful tool to infer microbe–microbe interactions [18], revealed that several bacterial taxa specifically associated with ‘Leccino’ showed potential interactions with *X. fastidiosa* [11]. Further studies could benefit from the evidence laid by Vergine et al. [11] and further develop potential mechanisms of colonization resistance associated with the microbiota of the ‘Leccino’ cultivar.

Plant resistance to pathogens is usually considered from the perspective of the host plant and is highly regarded when designing strategies for pathogen control. However, mechanisms that reduce pathogen virulence can be equally relevant for the control of pathogens affecting plants. In addition to mechanisms associated with direct competition of olive microbiota against *X. fastidiosa* itself, the microbiota associated with ‘Leccino’ and ‘Cellina di Nardò’ cultivars may also drive changes in *X. fastidiosa* virulence, as invading pathogens evolve in response to host microbiota [19]. Theory shows that pathogen virulence can be influenced by within-host microbial competition [20], as microbe-microbe interactions can decrease [21] or increase [22] pathogen virulence. For example, greenhouse experiments showed that different fungal endophyte strains reduced plant infestation by the aphid *Rhopalosiphum padi*, but the endophytes had no impact on levels of barley yellow dwarf virus (BYDV), an obligate aphid-transmitted virus [23]. Interestingly, BYDV virulence was reduced in endophyte-colonized plants, suggesting that host response modulation by endophytes could reduce pathogen virulence [23]. Similarly, the inoculation of the endophytic bacterium *P. phytofirmans* strain PsJN triggered the expression of the grapevine *PR-1* gene and reduced plant colonization by *X. fastidiosa*, and also decreased the PD symptoms caused by the pathogen in grapevine [16]. PR-1 activation is indicative of the induction of salicylic acid (SA)-mediated host defenses [8,16]. Interestingly, *X. fastidiosa* infection does not trigger SA-mediated defense pathways during early phases of infection, which is associated with higher virulence in plants [8]. Thus, by inducing the expression of innate immune resistance pathways in host plants, endophytic bacteria could prime the plant immunity to an early response against *X. fastidiosa*, in turn reducing virulence traits [16]. 

Host–microbiota balance is critical for host health, and infection-induced changes can result in dysbiosis with deleterious effects for animal hosts [19,24]. Vergine et al. [11] observed dysbiosis in the *X. fastidiosa*-infected ‘Cellina di Nardò’ cultivar, while ‘Leccino’ maintained a similar highly diverse microbiota in both *X. fastidiosa*-infected and -uninfected plants. In other resistant olive cultivar, FS17, a higher alpha diversity, was linked to a lower *Xylella* abundance, compared to susceptible cultivars [25]. *X. fastidiosa*-induced dysbiosis was characterized by reduced microbial diversity in susceptible olive trees [11]. Similarly, *X. fastidiosa* infection was correlated with a reduction in bacterial alpha diversity measures in almond trees [26]. Differences in the endophytic grapevine microbial community were found when severely symptomatic, mildly symptomatic, or asymptomatic PD phenotypes were considered [27]. In citrus, there were significant differences in endophyte incidence between leaves and branches and among healthy citrus-variegated chlorosis (CVC)-asymptomatic and CVC-symptomatic plants [28]. Within-plant–microbial interactions are so strong that the type and strength of pairwise connections can reliably predict the outcome of pathogen invasions [13]. Knowledge of the intricacies of microbe–microbe interactions within the microbiota may help to determine the key microbial players in *X. fastidiosa*–plant interactions, which may inform interventions such as synthetic microbial communities with broad plant-protective activities [29] for the control of *X. fastidiosa*.
